# RETRACTION: Antimicrobial and Controlled Release Studies of a Novel Nystatin Conjugated Iron Oxide Nanocomposite

**DOI:** 10.1155/bmri/9872508

**Published:** 2026-08-12

**Authors:** BioMed Research International

RETRACTION: S. H. Hussein‐Al‐Ali, M. E. El Zowalaty, A. U. Kura, B. Geilich, S. Fakurazi, T. J. Webster, M. Z. Hussein, “Antimicrobial and Controlled Release Studies of a Novel Nystatin Conjugated Iron Oxide Nanocomposite,” *BioMed Research International*, 2014, 651831, https://doi.org/10.1155/2014/651831

The above article, published online on 12 May 2014 in Wiley Online Library (wileyonlinelibrary.com), has been retracted by agreement between the journal Editor‐in‐Chief, Yoel Klug; and and John Wiley & Sons Ltd. The retraction has been agreed due to concerns with the reported data. As reported on PubPeer [1], Table 2, which shows the percentage inhibition of nystatin chitosan magnetic nanocomposite against different microorganisms, contains several repeated numbers that are highly unlikely to have occured by chance, including the following:

• The standard errors for Nys‐CS‐MNP 2 mg, CS 2 mg, MNPs 2 mg, CS‐MNP 1 mg for *S. aureus*, Nys‐CS‐MNP 2 mg for *P. aeruginosa*, MNPs 2 mg for *E. coli*, and CS 2 mg, CS‐MNP 1 mg, and CS‐MNP 2 mg for *C. albicans* are all 0.000;
• The standard errors for Nys‐CS‐MNP 1 mg and CS 2 mg for *S. aureus* are both 1.821;
• The standard errors for MNPs 1 mg and CS‐MNP 2 mg for *S. aureus* are both 0.910;
• The standard errors for Nys‐CS‐MNP 1 mg and CS‐MNP 2 mg for *P. aeruginosa* are both 0.081;
• The standard errors for CS 1 mg, MNPs 1 mg, and CS‐MNP 1 mg for *P. aeruginosa* are all 0.814;
• The standard errors for Nys‐CS‐MNPs 2 mg, CS 1 mg, and CS 2 mg for *E. coli* are all 0.431;
• The standard errors for MNPs 1 mg, CS‐MNP 1 mg, and CS‐MNP 2 mg for *E. coli* are all 4.314;
• The values ‐2.887 for MNPs 2 mg and 2.887 for CS‐MNP for *S. aureus* are identical, one negative and one positive;
• The values for CS 2 mg and CS‐MNP 1 mg for *C. albicans* are both 14.828.

The authors reported that these values are correct. They provided what they have stated are the underlying data in a PDF document. However, for all but one result, the mean for percentage inhibition exactly matches at least one of the three replicates, and for that exception, two of the three replicates are identical. The CFU/ml replicates that are used to calculate the percentage inhibition, when they are not identical, vary by the same increment, e.g., 340,000; 350,000; and 360,000; each value varies incrementally by exactly 10,000. Further, the replicates for several results, i.e., those with no standard error, are all identical.

The editor and the publisher have considered the evidence and determined that both the published data and the underlying data provided by the authors are highly unlikely to have occurred by chance, despite the authors’ insistence that they had provided accurate data both in the article and upon request to the publisher. The editor and publisher have lost confidence in the data and the reported results. Therefore, in keeping with COPE guidelines, the article must be retracted.

The authors disagree with the retraction and were disappointed at the length of time taken by the investigation, which began in 2020. The publisher acknowledges the long delay and also notes that additional time was spent communicating with the authors through their legal counsel, resulting in significant delays throughout the process.
